# Survival improvement of patients with *FLT3* mutated acute myeloid leukemia: results from a prospective 9 years cohort

**DOI:** 10.1038/s41408-023-00839-1

**Published:** 2023-05-05

**Authors:** Guadalupe Oñate, Marta Pratcorona, Ana Garrido, Alicia Artigas-Baleri, Alex Bataller, Mar Tormo, Montserrat Arnan, Susana Vives, Rosa Coll, Olga Salamero, Ferran Vall-Llovera, Antònia Sampol, Antoni Garcia, Marta Cervera, Sara Garcia Avila, Joan Bargay, Xavier Ortín, Josep F. Nomdedéu, Jordi Esteve, Jorge Sierra, Spanish Cooperative Group for the Study and Treatment of Acute Leukemias and Myelodysplasias (CETLAM)

**Affiliations:** 1 Hospital de la Santa Creu i Sant Pau. Institut d’investigació Biomèdica Sant Pau (IIB SANT PAU) Department of Medicine, Universitat Autonoma of Barcelona, Barcelona, Spain; 2 Hospital Clinic. August Pi i Sunyer Biomedical Research Institute (IDIBAPS), University of Barcelona, Barcelona, Spain; 3 Hospital Clinico Universitario, Biomedical Research Institute INCLIVA, Valencia, Spain; 4 Institut Catala d’Oncologia, Hospital Duran i Reynals, Institut d’Investigacio Biomèdica de Bellvitge (IDIBELL), L’Hospitalet de Llobregat, University of Barcelona, Barcelona, Spain; 5 Institut Catala d’Oncologia, Hospital Germans Trias i Pujol. Josep Carreras Leukemia Research Institute, Badalona, Universitat Autonoma of Barcelona, Barcelona, Spain; 6 Institut Català d’Oncologia, Hospital Josep Trueta, Girona, Spain; 7 Hospital Universitari Vall d’Hebron and Institute of Oncology (VHIO), Universitat Autonoma of Barcelona, Barcelona, Spain; 8 Hospital Universitari Mutua de Terrassa, Barcelona, Spain; 9 Hospital Universitari Son Espases, Palma de Mallorca, Spain; 10 Hospital Arnau de Vilanova, Lleida, Spain; 11 Institut Catala d’Oncologia, Hospital Joan XXIII, Tarragona, Spain; 12 Hospital del Mar, Barcelona, Spain; 13 Hospital Son Llatzer, Palma de Mallorca, Spain; 14 Hospital Verge de la Cinta, Tortosa, Spain

**Keywords:** Acute myeloid leukaemia, Translational research, Acute myeloid leukaemia

## Abstract

Midostaurin added to intensive chemotherapy is the standard of care for acute myeloid leukemia (AML) with *FLT3* mutations (*FLT3*mut). We analyzed the impact of midostaurin in 227 *FLT3*mut-AML patients included in the AML-12 prospective trial for fit patients ≤70 years (#NCT04687098). Patients were divided into an early (2012–2015) and late (2016–2020) cohorts. They were uniformly treated except for the addition of midostaurin in 71% of late group patients. No differences were observed in response rates or the number of allotransplants between groups. Outcome was improved in the late period: 2-year relapse incidence decreased from 42% vs 29% in early vs late group (*p* = 0.024) and 2-year overall survival (OS) improved from 47% vs 61% (*p* = 0.042), respectively. The effect of midostaurin was evident in *NPM1*mut patients (*n* = 151), with 2-yr OS of 72% (exposed) vs 50% (naive) patients (*p* = 0.011) and mitigated *FLT3*-ITD allelic ratio prognostic value: 2-yr OS with midostaurin was 85% and 58% in low and high ratio patients (*p* = 0.049) vs 67% and 39% in naive patients (*p* = 0.005). In the wild-type *NPM1* subset (*n* = 75), we did not observe significant differences between both study periods. In conclusion, this study highlights the improved outcome of *FLT3*mut AML fit patients with the incorporation of midostaurin.

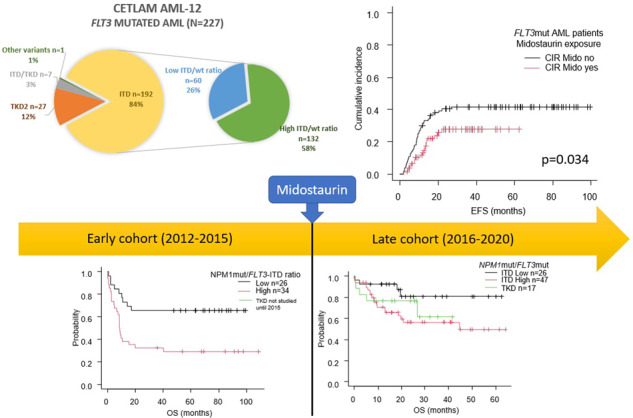

## Introduction

The treatment of patients with acute myeloid leukemia (AML) is rapidly evolving due to the advances in targeted therapy, risk-adapted protocols and measurable residual disease (MRD) guided decisions [1–4]. The implementation of molecular techniques with high sensitivity in everyday practice is now essential to accurately classify the different AML entities and to identify potential therapeutic targets in the AML cells. Therefore, a comprehensive molecular characterization of the disease is mandatory both at diagnosis and in the relapse and refractory setting for optimal treatment choice [5, 6].

FMS-like tyrosine kinase 3 (*FLT3*) is one of the most frequently mutated genes in AML with an incidence of around 30% and is generally associated with a negative outcome [7]. The most frequent mutations of *FLT3* are the internal tandem duplication (*FLT3*-ITD) near the juxta-membrane domain and point mutations of the TKD2 domain (*FLT3-*TKD), with an incidence of 22% and 8% respectively [8]. *FLT3* mutations (*FLT3*mut) result in the constitutive activation of the FLT3 receptor and the continuous transduction of pro-survival and proliferative signals via the RAS/MAPK, JAK/STAT5 and PI3K/AKT pathways [9].

The prognosis of patients harboring *FLT3*-ITD depends on several variables, such as the allelic ratio of the mutation, the presence of determinant co-mutations like *NPM1*, or the insertion site [10–14]. Regarding allelic ratio, several studies, including the National Comprehensive Cancer Network (NCCN) guidelines and the European LeukemiaNet 2017 classification (ELN-17) [15] emphasized its relevance in risk assessment. However, there is some controversy regarding the specific *FLT3*-ITD allelic threshold that can accurately divide high- and low-risk patients as well as concerns regarding the reproducibility of the technique. These are some of the reasons which led the experts to remove the modulating effect of *FLT3*-ITD allelic ratio in the latest prognostic classification of the ELN for AML (ELN-22) [16]. Anyhow, *FLT3*-ITD has in most instances a strong adverse impact and consequently, allogeneic hematopoietic cell transplantation (alloHCT) remains the recommended post-remission treatment in fit patients. Nonetheless, the indication of alloHCT in AML with *FLT3*mut might be redefined in the future given the potential benefit of *FLT3* inhibitors.

Midostaurin is a first-generation type 2 FLT3 inhibitor, with multi kinase inhibitory effect over various protein kinases such as FLT3, KIT, or PDGFR. The RATIFY phase III trial demonstrated a significant improvement in overall survival (OS) and event free survival (EFS) with the addition of midostaurin to standard 7 plus 3 chemotherapy in fit *FLT3*mut AML patients [17]. Based on this finding midostaurin was approved by the US Food and Drug Association and the European Medicines Agency in 2017 for all adult patients with AML with *FLT3* mutations.

A post-hoc analysis of the RATIFY trial demonstrated the benefit of midostaurin in reducing relapse risk in all ELN-17 prognostic categories [18]. However, the study was restricted to patients up to 59 years old. To date, only a few prospective phase II trials included patients older than 60 years who were intensively treated and also received midostaurin [19, 20]. These studies showed the feasibility of this combination in old fit patients as well as an overall outcome improvement compared to historical cohorts. Additionally, these studies also demonstrated the safety of midostaurin in the setting of post-alloHCT maintenance.

In 2016 the Spanish CETLAM group (Grupo cooperativo de estudio y tratamiento de las leucemias agudas y mielodisplasias) incorporated midostaurin to the therapy protocol for fit adults with AML and *FLT3* mutations. Patients were treated according the CETLAM AML-12 phase II trial (#NCT04687098). This included intensive chemotherapy (CT) induction followed by a consolidation approach within a risk-adapted post-remission strategy, with high-dose cytarabine or alloHCT according to the genetic risk at diagnosis and the persistence of MRD after treatment. This report focuses on the outcomes of *FLT3*mut patients up to 70 years before and after the introduction of midostaurin.

Two recent studies have analyzed the resulting effect of the incorporation of midostaurin [19, 21]. Up to the best of our knowledge, this constitutes the first study to address the issue in a patient population up to 70 treated with an homogenous chemotherapy backbone and a subsequent transplant decision based on a risk adapted criteria

## Subjects and methods

### Patients and samples

The series included patients with 18–70 years-old diagnosed with de novo AML and eligible for intensive chemotherapy within the CETLAM AML-12 phase II trial (#NCT04687098). Patients were treated at 15 academic centers in Spain between January 2012 and December 2020. Since 2016, patients with *FLT3*mut could receive midostaurin added to the therapy as part of the AML-12 trial. The trial was approved by the IRB boards at the participating institutions and by the health authorities and was conducted in accordance with the Declaration of Helsinki. Informed consent for both bone marrow analyses and treatment was obtained in all cases.

In order to analyze the impact of midostaurin on the outcome, we considered two study cohorts: “early cohort” (treated between 2012 and 2015) and “late cohort” (2016–2020). All patients received the same CT protocol except for the addition of midostaurin since 2016 in most *FLT3*mut patients. The criteria to indicate alloHCT in first CR (CR1) were unchanged throughout the study period.

Full protocol details are available at www.clinicaltrials.gov and are detailed in supplemental material. In brief, all patients enrolled in the AML-12 trial received induction chemotherapy with idarubicin and cytarabine. The definition of complete remission (CR), overall survival (OS), event-free survival (EFS) and cumulative incidence of relapse (CIR) followed the recommended ELN criteria [22]. Response assessment was performed after one cycle: patients with a partial response received a second induction cycle; if first complete remission with or without complete hematological recovery (CR or CRi) [23] was achieved, they proceeded with first consolidation with high dose cytarabine (HDAC) according to the classical CALGB scheme [24]. Risk stratification was based on ELN recommendations (ELN-2010 [23] until the introduction of ELN-2017 [15]) but patients with *NPM1*mut and *FLT3*-ITD low allelic ratio were considered favorable since the beginning of the protocol. Favorable-risk patients completed two more HDAC courses and continued with MRD monitoring, while intermediate or adverse patients were intended for alloHCT in CR1 after one or two HDAC consolidations. MRD was assessed in bone marrow samples after each treatment cycle by either multiparameter flow cytometry or molecular monitoring of *NPM1* transcripts (as described by Gorello et al.) and, in a few cases, of rearrangements *RUNX1::RUNX1T1* and *CBF::MYH11*.

### Genomic analysis

All analysis were performed at diagnosis on bone marrow samples. Molecular testing of *NPM1* mutation was studied as previously described [25]. All patients were tested for *FLT3* internal tandem duplication (*FLT3*-ITD) [26] and classified according to its ITD/wt allelic ratio following previous recommendations (<0.5 low ratio, *FLT3*low; and ≥0.5 high ratio, *FLT3*high) [11, 15]. *FLT3*-TKD mutations were not routinely reported until 2015. Next-generation sequencing with a targeted panel of 42 genes was introduced in 2017 as part of the protocol diagnostic work-up allowing the detection of additional, less frequent *FLT3* mutations (Supplemental Fig. 2).

### Statistical analysis

The analysis of the relationship between categorical variables was performed using the Chi-square test or the Fisher exact test. Differences between groups for continuous variables were studied by means of the independent-samples t-test or Mann-Whitney U. All tests were two sided and considered significant if *p* < 0.05. Follow-up duration was calculated with the inverted Kaplan-Meyer method [27, 28]. OS and EFS were studied with the Kaplan-Meier method, whereas cumulative incidences were calculated to estimate relapse risk and non-relapse mortality (NRM) considering death in remission and relapse as competitive end-points, respectively. Differences between groups were assessed with the log-rank test (OS, EFS) and the Gray test (CIR, NRM) and were considered significant when *p* < 0.05. Cox-proportional hazard regression was used for multivariable analysis. AlloHCT was analyzed as a time-dependent variable. All statistical analyses were performed with the SPSS software (Version 26, IBM, Armonk, NY, USA) and R statistics (Version 4.0.2, R Foundation for Statistical Computing, Vienna, Austria).

## Results

### Characteristics of patients

*FLT3* mutations were detected in 227 cases (25%) out of 906 patients included in the AML-12 protocol between 2012 and 2020, and constitute the study population. Median follow up is 42 months (95%CI 35–49). The early cohort included 94 patients whereas the late included 133. Characteristics of these patients, considering both time cohorts (2012–2015 and 2016–2020) are summarized in Table 1. Cytogenetics was available in 93% of cases. No significant differences were observed between the two study cohorts including the ELN prognostic category, prevalence of *NPM1* co-mutation or the proportion of patients harboring a *FLT3*-ITD with a high allelic ITD/wt ratio, with the exception of the virtual absence of TKD sole mutations in the early cohort. Since the analysis of *FLT3*-TKDs started in 2015, only 2 patients from the early cohort were identified compared to 25 from the late period cohort. Only one less frequent *FLT3* mutation (F594I) was detected in a patient with a complex karyotype.

**Table 1:** Characteristics of patients treated according CETLAM AML-12 protocol and with an identified *FLT3* mutation.

CETLAM AML-12 patients < 70 years with *FLT3*mut (*n* = 227)			
	2012–2015 *n* = 94	2016–2020 *n* = 133	*P*
Female gender *n* (%)	52 (55)	75 (56)	0.9
Median age (range)	54 (21–70)	55 (20–70)	0.65
<60 years *n* (%)	65 (69)	87 (65)	
≥60 years *n* (%)	29 (31)	46 (35)	
ECOG 0–1 *n* (%)	77 (82)	102 (77)	0.3
Median WBC ×10^9^/L (range)	53 (1.6–314)	45 (0.42–395)	0.9
Median BM blasts % (range)	80% (21–100)	80% (21–100)	0.44
Cytogenetics prognostic category^&^ *n* (%)			0.9
Favorable	4 (4)	4 (3)	
Intermediate	82 (88)	111 (83)	
Adverse	4 (4)	5 (4)	
No metaphases	4 (4)	13 (10)	
*FLT3* mutations n (%)			
*FLT3*-ITD	92 (98)	100 (75)	0.54
Low ratio	33 (36)	27 (31)	—
High ratio	59 (64)	73 (69)	—
*FLT3*-TKD^#^	2 (2)	25 (19)	—
*FLT3*-ITD with TKD^#^	0	7 (5)	
Other	—	1 (1)	
Concomitant *NPM1*mut *n* (%)	61 (65)	90 (68)	0.7
ELN-17 prognostic categories			0.8
Favorable	30 (32%)	45 (34%)	
*RUNX1::RUNX1T1*	3	3	
*CBFB::MYH11*	1	1	
*NPM1*mut*/FLT3*low	25	25	
*NPM1*mut*/FLT3-*TKD	1	16	
Intermediate	38 (40%)	57 (43%)	
*NPM1*mut*/FLT3*high	34	47	
*NPM1*wt*/FLT3*low	3	5	
*NPM1*wt*/FLT3-*TKD	1	5	
Adverse	26 (28%)	31 (23%)	
*NPM1*wt*/FLT3*high	23	24	
Other*	3	7	
CR rate *n* (%)	73 (78)	109 (82)	0.7
AlloHCT *n* (%)	53 (56)	82 (62)	0.6
CR1	39 (41)	64 (48)	
CR2	6 (6)	9 (7)	
Active disease	5 (5)	7 (5)	
Unknown status	3 (5)	2 (2)	

*AlloHCT* allogeneic hematopoietic cell transplantation, *BM* bone marrow, *CR* complete remission, *ELN* European LeukemiaNet, *CR1* first complete remission, *CR2* second complete remission, *NA* not applicable, *OS* overall survival, *WBC* White blood count ^&^Cytogenetic risk defined according to MRC/NCRI recommendations (Grimwade et al. Blood 2010) ^#^TKD mutations were not routinely analyzed until 2015

*Two cases in the early period presented with t(6;9) while the other had a complex karyotype (CK), in the late period 2 patients had CK, 1 a t(6;9), 2 patients harbored mutated *TP53*, and 2 cases a mutated *RUNX1* with an absence of *NPM1*mut

### Impact of midostaurin use

**Fig. 1:**
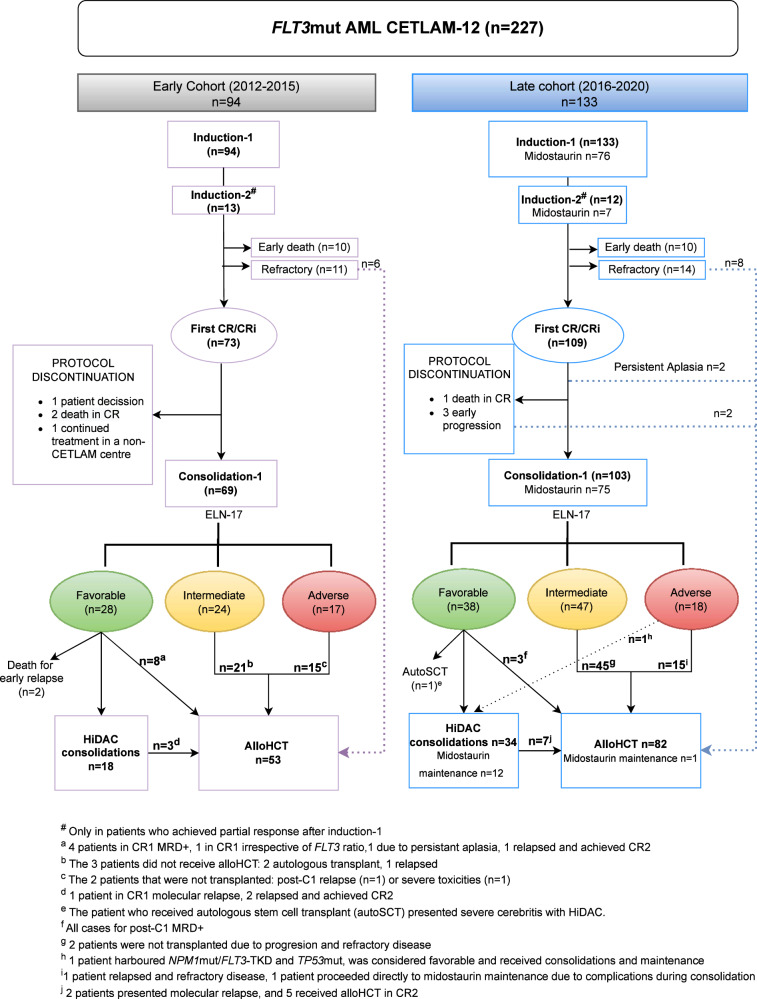
CONSORT diagram of patients in both time periods. AlloHCT: allogeneic stem cell transplant, AutoSCT: autologous stem cell transplant, C-1: first consolidation, CR1: first complete remission, CR2: second complete remission, HiDAC: high-dose cytarabine, MRD + : positive measurable residual disease.

Seventy-one per cent (94/133) of patients from the late cohort received midostaurin at some point during frontline treatment (Fig. 1): 71 patients with an ITD (22 *FLT3*low and 49 *FLT3*high), 20 with TKD, and 3 with other *FLT3*mut. The onset of the treatment was during induction-1 in most patients (81%), although treatment start was deferred in the remaining patients due to an administrative gap between initial request and real availability of the drug during the initial period, which required compassionate request on a case-to-case basis. Fourteen patients of this group received midostaurin as maintenance, mainly (*n* = 12) due to a non-transplant allocation given a favorable genetic risk according to ELN-2017. Maintenance was administered in one patient with *NPM1*mut/*FLT3*high during the posttransplant period, whereas one unfavorable patient with *NPM1*wt/*FLT3*high was not eligible for alloHCT after chemotherapy, and started maintenance following consolidation-1.

Response rate after induction was similar in both time cohorts (CR/CRi rate 78% (*n* = 73) in the early group and 82% (*n* = 109) in the late period group; *p* = ns). Treatment failure was related to refractory disease (12% in the early vs 11% in the late cohort, p=ns) or death in aplasia (11% vs 8%, respectively p=ns). An early relapse in the first 3.5 months (median time from CR to transplant) occurred in 9 patients between 2012 and 2015 and in 5 patients in the latter period 2016–2020 (*p* = 0.089). AlloHCT was performed in a similar proportion of patients in both time cohorts (in 56% (*n* = 53) and 62% (*n* = 82) of patients, respectively [*p* = ns]). For patients achieving CR1, the alloHCT rate was 64% (*n* = 47) in the early group and 68% (*n* = 74) in the late group. In the overall cohort, MRD persistence following first consolidation was associated with higher relapse-risk (2 yr 48 ± 15% vs 28 ± 8% p = 0.021) and a trend towards worse survival (2 yr 57 ± 8% vs 70 ± 4% *p* = 0.055, Supplemental Fig. 3). However, we did not observe a significant association between MRD negativity and time period (73% MRD negative in early and late cohort) or midostaurin exposure (74% in both midostaurin naive and exposed patients).

**Fig. 2:**
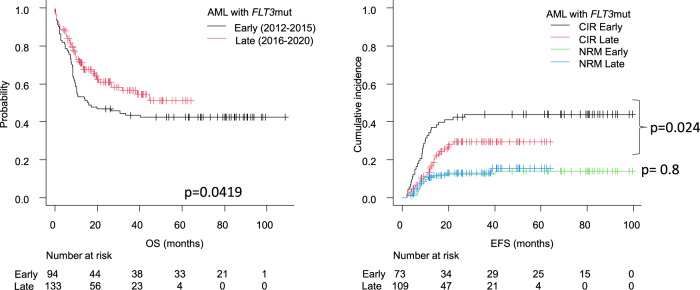
Outcome of CETLAM AML-12 *FLT3*mut patients (*n* = 227), according to treatment period. CIR: cumulative incidence of relapse, EFS: event-free survival, NRM: non-relapse mortality, OS: overall survival.

Early cohort *FLT3*mut patients presented with a significantly higher CIR compared to the late cohort (2-year (2 yr) CIR of 42 ± 11% vs 29 ± 10% for early and late group, respectively; *p* = 0.024), without differences in NRM (2 yr NRM of 12% vs 13% respectively; *p* = 0.8; Fig. 2). This translated into an improved outcome of late cohort patients, regarding EFS (2-yr 37 ± 5% vs 50 ± 5% for early and late groups; *p* = 0.021) and OS (2-yr of 47 ± 5% vs 61 ± 5%, in each group, respectively; *p* = 0.042, Fig. 2).

**Fig. 3:**
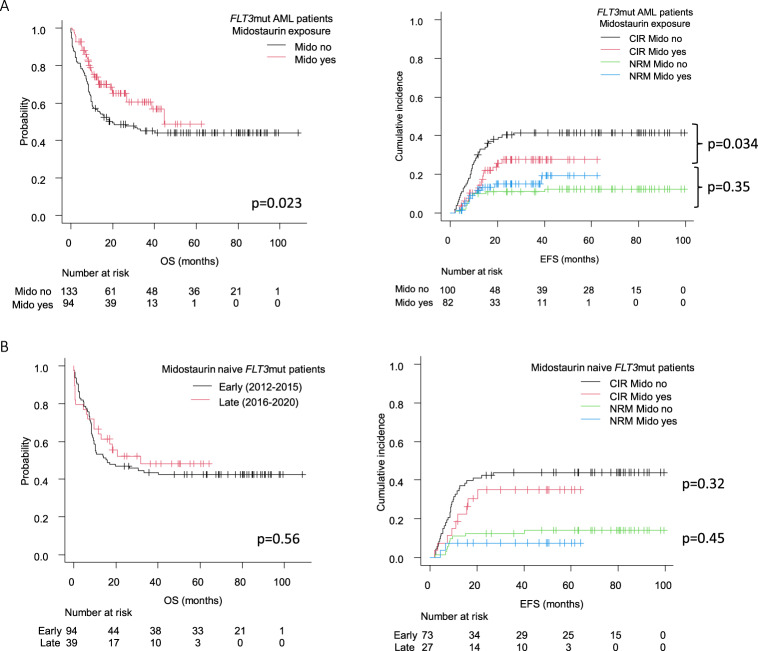
Survival outcome of midostaurin use in AML-12 *FLT3*mut patients. **A** OS, CIR and NRM of the overall cohort according to midostaurin exposure. **B** OS, CIR, and NRM in both time periods among patients who did not receive midostaurin OS: overall survival, CIR: cumulative incidence or relapse, NRM: non-relapse mortality.

To establish the influence of different variables on survival, we performed univariate analysis for all *FLT3*mut patients, and found that leucocyte count (WBC) at diagnosis, treatment period, ELN-17 *FLT3* categories, and the presence of *NPM*1mut significantly impacted on OS (supplemental Table 1). The HR for OS of midostaurin administration was 0.62; 95%CI 0.41–0.94; *p* = 0.024. Thus, midostaurin led to improved survival in the whole cohort by decreasing relapse incidence (Fig. 3A) with a 2 yr RR of 40 ± 9% vs 28 ± 10% (*p* = 0.034) for naive and exposed patients, and a 2 yr OS of 49 ± 4% vs 65 ± 5% (*p* = 0.023) respectively. It is noteworthy that patients in the late cohort who did not receive midostaurin had very similar survival to those from the early cohort (Fig. 3B).

In a multivariate model including age, midostaurin use, WBC at diagnosis and ELN-17 prognostic categories, midostaurin maintained its independent prognostic value both for OS and EFS with a HR for OS of 0.55 (95%CI 0.36–0.85; *p* = 0.007), and for EFS of 0.51 (0.34–0.76; *p* = 0.001, Table 2), for patients exposed to the drug.

**Table 2:** Multivariate Cox-proportional hazard regression in each *FLT3*mut subset.

	OS			EFS		
FLT3*mut*	HR	95% CI	*p*	HR	95% CI	*p*
WBC	1.002	1–1.005	0.025	1.003	1.001–1.005	0.003
Age <60 years	0.61	0.41–0.91	0.016	0.61	0.42–0.88	0.008
ELN-17 subcategories						
intermediate risk (vs fav)	2.36	1.38–4.04	0.002	2.21	1.38–3.55	0.001
adverse risk (vs. fav)	3.47	1.99–6.04	<0.001	3.19	1.95–5.23	<0.001
Midostaurin	0.55	0.36–0.85	0.007	0.51	0.34–0.76	0.001
*NPM1*mut *FLT3*mut						
WBC	1.002	0.999–1.005	0.25	1.003	1.000–1.006	0.027
Age<60 years	0.50	0.29–0.85	0.011	0.53	0.33–0.87	0.012
ELN-17 interm vs fav risk	2.39	1.34–4.24	0.003	2.13	1.28–3.53	0.003
Midostaurin	0.40	0.22–0.72	0.002	0.34	0.20–0.59	<0.001
*NPM1*wt *FLT3*mut						
WBC	1.005	1.002–1.009	0.006	1.005	1.002–1.009	0.002
Age < 60 years	0.73	0.38–1.43	0.4	0.64	0.35–1.17	0.15
ELN-17 subcategories						
intermediate risk (vs fav)	1.93	0.38–9.91	0.43	2.02	0.52–7.89	0.3
adverse risk (vs. fav)	3.65	0.86–15.5	0.08	3.12	0.94–10.4	0.063
Midostaurin	1.07	0.55–2.08	0.9	1.09	0.59–2.03	0.8

*CI* confidence interval, *EFS* event free survival, *HR* Hazard ratio, *OS* overall survival, *WBC* leucocyte count

### Impact of midostaurin in AML with *NPM1* mutation

**Fig. 4:**
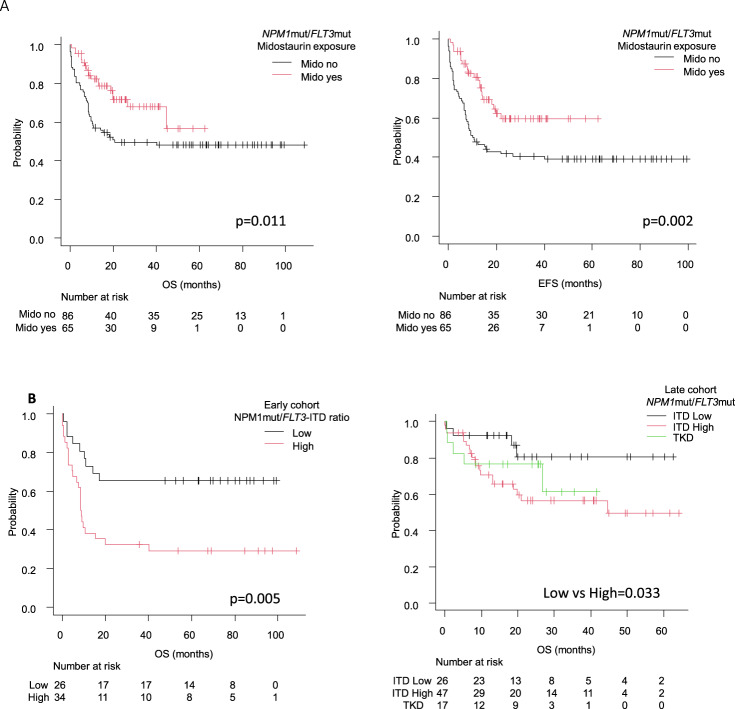
Outcome of AML patients with FLT3 mutations and co-mutated NPM1. **A** OS and EFS of patients according to midostaurin exposure. **B** OS of each *NPM1*mut/*FLT3*mut subset in the early (left) and the late groups. *FLT3-*TKD mutations were not available in the early period.

A total of 151 patients harbored *NPM1*mut and *FLT3*mut simultaneously. There was an improved outcome of AML-*NPM1*mut patients who received midostaurin (*n* = 65), with a 2-yr EFS of 42 ± 5% vs 60 ± 7% for naive vs exposed patients (*p* = 0.002) and 2-yr OS of 50 ± 5% vs 72 ± 6% respectively (*p* = 0.011) (Fig. 4A). Moreover, the HR for OS of midostaurin exposure in the univariate analysis was 0.50 (95%CI 0.29–0.86, *p* = 0.013), a finding that was maintained in the multivariate analysis both for OS (HR 0.40 (0.22–0.72; *p* = 0.002) and EFS (HR 0.34 (0.20–0.59; *p* < 0.001, Table 2).

**Fig. 5:**
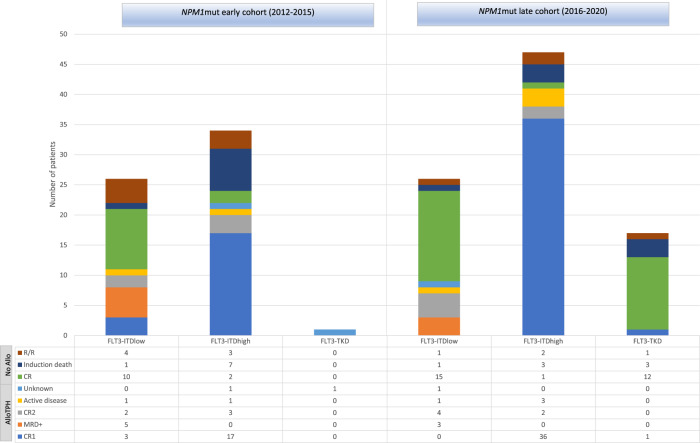
Description of the disease status of *NPM1*mut/*FLT3*mut patients in each time period and *FLT3* subset. In allogeneic stem cell transplant (AlloHCT) recipients, pre-transplant evaluation is shown, in non-allotransplanted patients the last available evaluation is informed. CR complete remission (first: CR1, second: CR2), MRD + positive measurable residual disease, R/R relapse or refractory disease.

The allelic ratio of *FLT3*-ITD retained its prognostic value in both time cohorts (Fig. 4B). Thus, the 2-yr OS of patients treated during 2012 and 2015 was 65 ± 9% for *FLT3*low (*n* = 26) vs 32 ± 8% for *FLT3*high (*n* = 34; *p* = 0.005) whereas in the late period the adverse prognosis of *FLT3*-ITD was highly mitigated with a 2 yr OS of 81 ± 9% for *FLT3*low patients (*n* = 26) and 57 ± 8% for the *FLT3*high patients (*n* = 47) (*p* = 0.033). Similar findings were seen in terms of EFS (Supplemental Fig. 4) and when midostaurin exposure was directly contrasted (2-yr OS with midostaurin was 85% in low and 58% in high ratio patients (*p* = 0.049) vs 67% and 39% in naive patients; *p* = 0.005). On the other hand, *NPM1*mut/*FLT3-*TKD patients (*n* = 18) presented with a 2-yr OS of 72 ± 10%.It is worth mentioning that *NPM1*mut/*FLT3*low patients from the late cohort had a strikingly favorable outcome with a 2 yr OS of 81%, and more than half of those patients did not receive an alloHCT (64%, 16 out of 25 patients alive after induction). Among the 9 patients that did undergo alloHCT, 3 were performed due to positive MRD, 4 in CR2 status, 1 had active disease, and the disease status of the remaining patient was unknown (Fig. 5). Moreover, midostaurin was administered to 83% of patients from the late cohort that presented with non-high-risk disease (*NPM1*mut with *FLT3*low or TKD) who were never transplanted, and their 2-yr CIR was 5% in the late group (*n* = 29) vs 29% in the early group (*n* = 14, *p* = 0.022) with similar NRM (7%) in both cohorts (Supplemental Fig. 5).

Seventy-eight percent of *NPM1*mut/*FLT3*high patients (63 out of 81) received an alloHCT in the overall cohort, with 84% of them transplanted in CR1 (Fig. 5). We observed a significantly lower CIR of *NPM1*mut/*FLT3*high patients who received an alloHCT in the late group (71% of which had received midostaurin) in contrast to the early group (2-yr 55% vs 30%; *p* = 0.044) with no differences in NRM (*p* = 0.2; Supplemental Fig. 6).

### Impact of midostaurin in AML with wild-type *NPM1*

Seventy-five patients presented with *FLT3*mut without mutation of *NPM1*, 33 and 42 from the early and late periods, respectively. The main clinical characteristics were balanced between cohorts (supplemental Table 2). There were no significant differences in karyotype distribution which was mostly of intermediate risk (76%) according to the Medical Research Council definition [29]. Nine percent of patients presented with CBF rearrangements, and *FLT3*-ITD allelic ratio was high in 78% of ITD patients in both cohorts. Also, 8 out of the 9 patients who presented *FLT3-*TKD mutations were from the late cohort and none of them were associated to a favorable karyotype.

Fifty-four patients (72%) achieved CR1 following induction and alloHCT was performed in 58% (19/33) and 74% (31/42) patients from the early and late cohort respectively (*p* = 0.3; Supplemental Fig. 7). Twenty-nine out of 42 late cohort patients received midostaurin (69%). Although the cohort size was limited, in the absence of *NPM1*mut we found no significant survival improvement with the use of midostaurin with a median EFS of 10 months for naive and exposed patients (*p* = 0.9) and a 2-yr OS of 48 ± 7% vs 51 ± 10% in the same groups respectively (*p* = 0.9, Supplemental Fig. 8), with a HR for OS (midostaurin) of 0.95 (95%CI 0.51–1.79; *p* = 0.9). A trend towards higher relapse rate was seen in the early group (2-yr CIR 54 ± 19% vs 33 ± 17% in the early and late groups; *p* = 0.14, Supplemental Fig. 9), but it did not translate into a significant survival improvement possibly due to the limited cohort size and a higher NRM of late cohort patients in this subgroup (NRM at 2 yr 8% vs 28% in the early and late groups, respectively *p* = 0.10). The global outcome of this molecular subgroup of patients remained poor throughout the protocol with a median EFS around 10 months in both groups, and a 2-yr OS of 49 ± 9% vs 48 ± 8% in the early and the late groups (*p* = 0.9, Supplemental Fig. 10). In the absence of *NPM1*mut, the small subset of non-ITD patients (*n* = 10: 9 TKDs and 1 other mutation) maintained their favorable outcome with a 2-yr OS of 73 ± 16%.

## Discussion

This study confirms the benefit of midostaurin in patients with AML and mutations of *FLT3* eligible for intensive chemotherapy, a subgroup that includes 30% of adults with this disease and is associated with adverse outcomes [30–32]. This beneficial effect was observed in patients with an age up to 70 years, treated following the same therapeutic protocol with a post-remission strategy adapted to genetic risk. Moreover, the effect of midostaurin was mostly attributed to a decrease in relapse risk and was confirmed in a multivariate analysis.

In our experience, the prognostic improvement was predominantly observed in patients with *NPM1* co-mutation. Of note, the outcome of patients both with *NPM1*mut/*FLT3*-ITD low ratio with an OS of 81% at two years, and *NPM1*mut with *FLT3*-TKD mutations (2-year OS 71%) was remarkably improved in the most recent period 2016–2020, two subgroups without an initial alloHCT intention in CR1. Overall, our experience is consistent with the results of the RATIFY trial that provided the most solid data on the benefit of adding midostaurin to intensive front-line chemotherapy in *FLT3*mut AML. This worldwide trial randomized 717 *FLT3*mut patients to receive midostaurin or placebo with induction and consolidation CT and showed an improvement in survival in the group with FLT3 inhibitor in the whole series as well as in all the molecular subgroups. A few studies have validated this finding; Larson et al. reported a post-hoc analysis of the RATIFY trial emphasizing the benefit of midostaurin in decreasing the cumulative incidence of relapse (HR 0.71 (95% CI, 0.54–0.93); *p* = 0.01). The trial and the analyses included patients up to 59 years old [18]. The AMLSG 16–10 phase 2 study, showed the positive impact of midostaurin in 198 younger and 86 older (>60 years) patients treated between 2012 and 2016 and compared the outcomes with historical controls from 5 AMLSG trials between 1993 and 2008 [19, 20]. Recently, the MD Anderson group published a series showing the improvement in prognosis of patients with *FLT3*mut AML, mostly due to the implementation of targeted therapy with FLT3 inhibitors. In contrast to our study, they included patients mainly treated with sorafenib or quizartinib which are not commonly used as front-line therapy in this situation, and the report analyzed the addition of the FLT3 agents both in intensive and non-intensive combinations [21]. Differently from this, our study included only intensively treated patients and, as the AMLSG study, we analyzed patients up to the age of 70 years who were homogeneously treated with intensive CT and transplantation, the latter indicated based on risk assessment. It is important to emphasize that in our report 33% of the patients had an age between 60 and 70 years.

In the AML-12 trial the indication of alloHCT was defined according to risk at diagnosis and MRD evolution; in all intermediate and adverse patients the intention was to proceed to alloHCT unless major complications aroused during chemotherapy and in the favorable genetic category only patients in remission with persistent or reappearing MRD or those with cytological relapse were considered for alloHCT. All these interventional strategies did not allow, in our view, a fair assessment of the impact of alloHCT in the multivariable analysis. In fact, when we made an exploratory assessment of the impact of transplantation in the favorable ELN-17 subgroup (*NPM1*mut with *FLT3*low or TKD) and performed a Cox regression with transplantation as time-dependent covariate, a worse survival in transplant recipients was observed (HR for OS (Allo) 4.71 95% CI 1.31–16.95 *p* = 0.018) that we consider was attributable to the worse characteristics of transplanted cases (MRD positive or in relapse).

One of the limitations of our study is that we retrospectively compared two different study periods, and the favorable outcome of the late cohort might be influenced by other factors such as changes in alloHCT platforms or advances in support measures. However, during the period analyzed, neither chemotherapy strategies nor transplantation techniques substantially changed in our cooperative group and the only major advance was the introduction of midostaurin. Also, the outcomes of patients with AML that we treated with an identical protocol without *FLT3* mutations did not change between 2012 and 2020 (data not shown), and the OS improvement of *FLT3*mut patients was related to a decrease in their relapse risk. These facts reinforce that the improved outcome of *FLT3*mut-AML patients in recent years was due to the addition of this agent to the chemotherapy courses.

In accordance with previous CETLAM publications, we also performed molecular subgroup analyses. We, as the AMLSG, stratified patients according to the *FLT3*-ITD allelic ratio of 0.5. A prior study from our group revealed that this threshold had prognostic impact [11] and in a recent analysis we confirmed that this allelic ratio remained relevant regardless of the presence of *DNMT3A* co-mutation [4]. Of note, both studies analyzed *FLT3*mut patients treated before FLT3 inhibitors were available. Furthermore, a publication by Dohner et al. validating the ELN-17 classification in the RATIFY cohort showed that 0.5 was also the best discriminant value to define patients with different prognoses based on allelic ratio [12]. This differs from the original RATIFY trial that considered the 0.7 ratio cut-off. However, the limitations in the reproducibility of the technique to establish the ratio and the results from other investigators have led to the withdrawal of this aspect in the most recent ELN 2022 genetic classification of AML [16]. These ELN-2022 guidelines consider all *FLT3*-ITD patients as intermediate risk regardless of *NPM1* co-mutation or ITD allelic ratio and recommend alloHCT in CR1. The results from our study in patients with *NPM1*mut/*FLT3-*ITD low ratio challenges this recommendation; in our view, the results observed with consolidation CT only support continuing our current practice of delaying transplantation after a relapse, provided that there is no persistence of MRD. These patients had an outstanding OS since the introduction of midostaurin with a relapse incidence of less than 10% at 2 years. On the other hand, the results observed in the *NPM1*mut/*FLT3*-ITD high ratio who were candidates for alloHCT in CR1 also were significantly improved after midostaurin was introduced. In this regard, in this molecular category OS at 2 years was higher than 50% in the midostaurin era. Further analyses are needed to identify whether in a subgroup of these patients, allograft could be avoided or delayed to a later moment based on MRD absence and pattern of co-mutations. On the other hand, additional progress is needed in the *NPM1*wild/*FLT3*-ITD group, since midostaurin in our hands did not improve their outcome. This particular finding should be weighed carefully due to the limited size number. We analyzed a possible impact of midostaurin exposure in the pretransplant setting in *NPM1* mutated vs wild type and found that the median number of days in the first group was 25 vs 18 in the second. This difference, however, was not statistically significant. Finally, TKD mutations were only available in the late cohort, and therefore their outcome could not be comprehensively contrasted. Additional division of ITD/TKD subsets as well as the impact of other co-mutations resulted in too few patient in each subgroup to provide statistical significance in the current analysis and should be explored in larger studies.

We consider that studies as the one reported here are of value. Randomized clinical trials set to register novel agents in AML patients usually present strict inclusion criteria regarding performance status, organ functions and coexistence of infections at diagnosis, among others. Therefore, it is useful to confirm their results in less restrictive trials from academic groups such as ours that make much fewer limitations to inclusion concerning real-life high ECOG scores or active infections and accept adjustments of drugs for renal or liver abnormalities that would make the patients ineligible for trials promoted by corporate sponsors. The complementarity of registration trials, academic experiences and real data gives a more solid picture of the impact of new drugs to improve the prognosis of a rare and difficult disease such as AML.

In summary, this study highlights the positive change in prognosis of *FLT3*mut patients due to the association of CT and targeted therapy with midostaurin, in patients included in a prospective trial during 9 years comprising patients with an age up to 70 years and with a risk-adapted pre-defined post-remission alloHCT policy. Further research is needed in patients with AML and *FLT3*mut in the absence of *NPM1* mutation. It will also be of interest to know if the results reported here can be further improved with other FLT3 inhibitors.

## Supplementary information

revised supplemental material

## Data Availability

The datasets generated during and/or analyzed during the current study are available from the corresponding author on reasonable request.
