# Supplementary material for: Survival improvement of patients with FLT3 mutated acute myeloid leukemia: results from a prospective 9 years cohort
Source: Blood Cancer J. 2023 May 5;13(1):69. doi: 10.1038/s41408-023-00839-1 (PMC10162955; doi:10.1038/s41408-023-00839-1)
Supplement: Supplementary file 1 — revised supplemental material [file 41408_2023_839_MOESM1_ESM.docx]

SUPPLEMENTAL MATERIAL


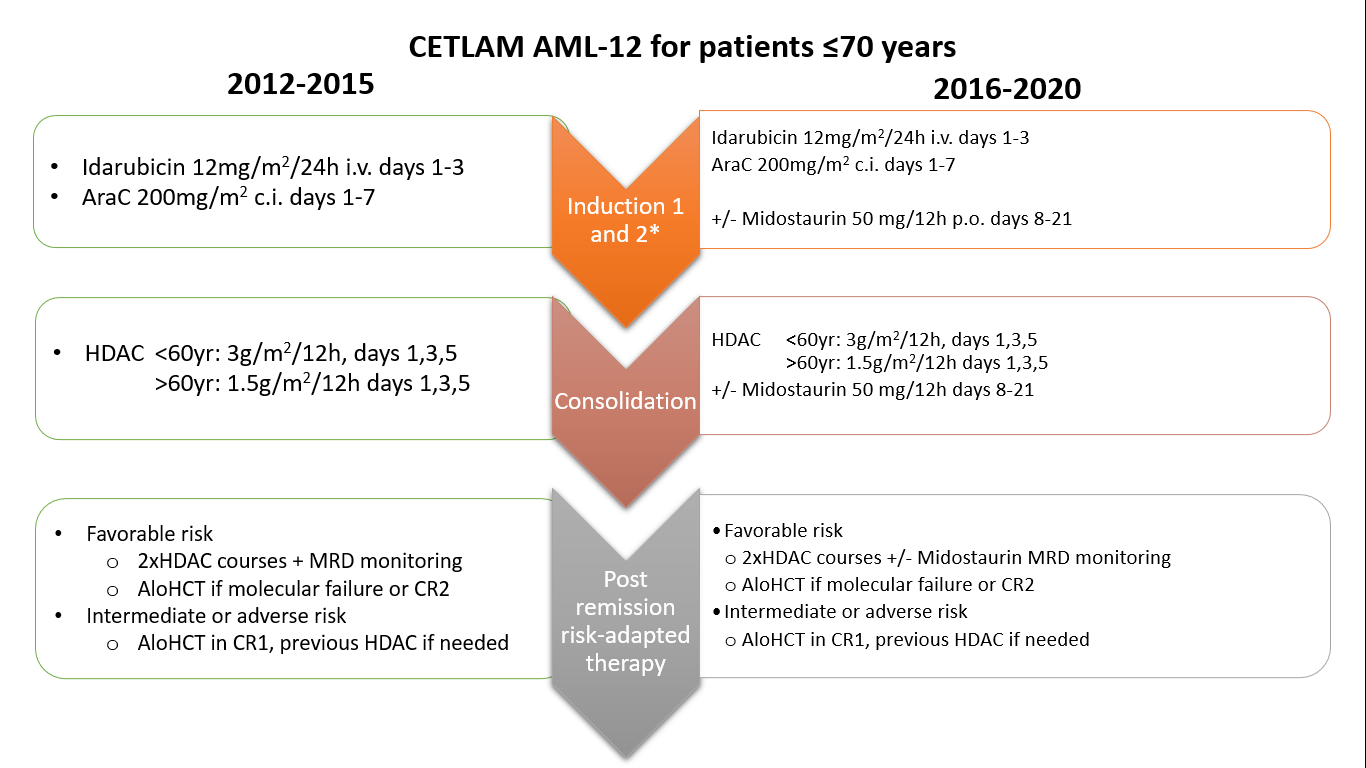
**Supplemental Figure 1:** CETLAM AML-12 intensive protocol algorithm.

*Induction-2 only administered in case of partial response

AlloHCT: allogeneic stem cell transplant, CR1: first complete response, CR2: second complete response, HDAC: High dose cytarabine, MRD: Measurable residual disease, Yr: years

**Supplemental figure 2**: *FLT3* mutation distribution in the whole cohort (upper figure) and co-mutational pattern according to next generation sequencing results and ELN-17 categories. NGS was only included in the protocol from 2017 and was therefore performed in 67 patients from the late period (lower figure).

*****

**Supplemental Figure 3**: Impact of MRD persistence after consolidation-1 in the overall cohort of *FLT3*mut patients by overall survival (left) and cumulative incidence of relapse (right).


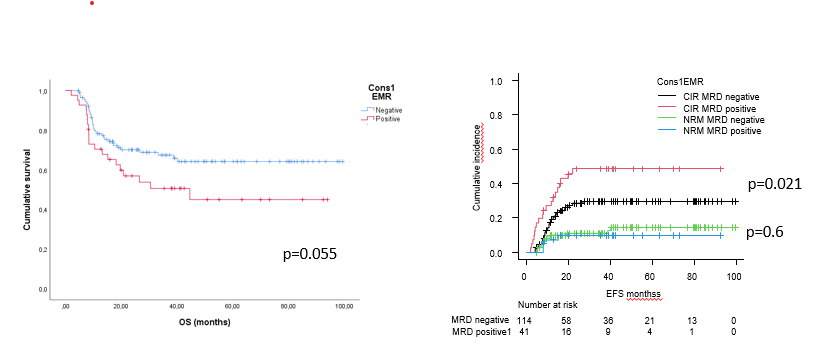


**Supplemental Table 1:** Univariate Cox-regression for OS of all *FLT3*mut patients

|  | **HR for OS** | **95,0% CI** | | **p** |
| --- | --- | --- | --- | --- |
|  |  | **Inferior** | **Superior** |  |
| **Gender (male)** | 1.18 | 0.81 | 1.72 | 0.4 |
| **Age<60** | 0.68 | 0.46 | 1.005 | 0.053 |
| **Treatment Period** Early | 1.49 | 1.01 | 2.18 | 0.043 |
| **ELN-17** intermediate vs fav | 2.51 | 1.48 | 4.27 | 0.001 |
| adverse vs fav | 3.6 | 2.07 | 6.26 | <0.001 |
| **WBC at diagnosis** | 1.003 | 1.001 | 1.005 | 0.015 |
| **Bone marrow Blasts** | 1.001 | 0.991 | 1.010 | 0.9 |
| ***NPM1*mut** | 0.67 | 0.46 | 0.99 | 0.046 |
| **FLT3**-ITD high ratio | 2.71 | 1.64 | 4.49 | <0.001 |
| **Midostaurin** | 0.62 | 0,41 | 0,94 | 0.024 |
| Post-induction result (refractory) | 3.27 | 1.92 | 5.58 | <0.001 |
| **Nº cycle to CR (1)** | 0.72 | 0.36 | 1.45 | 0.4 |

**Supplemental Figure 4**: Event free survival of early (left) and late (right) *NPM1*mut patients according to *FLT3*-ITD allelic ratio


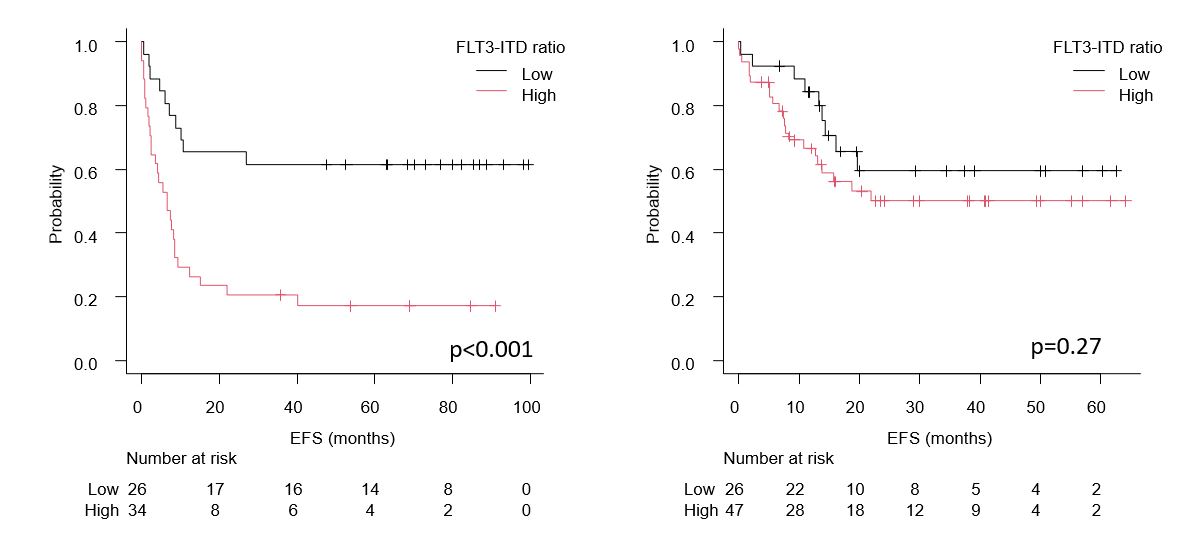


**EARLY COHORT**

**LATE COHORT**

**Supplemental figure 5:** Outcome of non-transplanted *NPM1*mut *FLT3-*ITD low ratio patients in each time period: Overall survival (upper figure) and cumulative incidence of relapse and non-relapse mortality (lower figure)


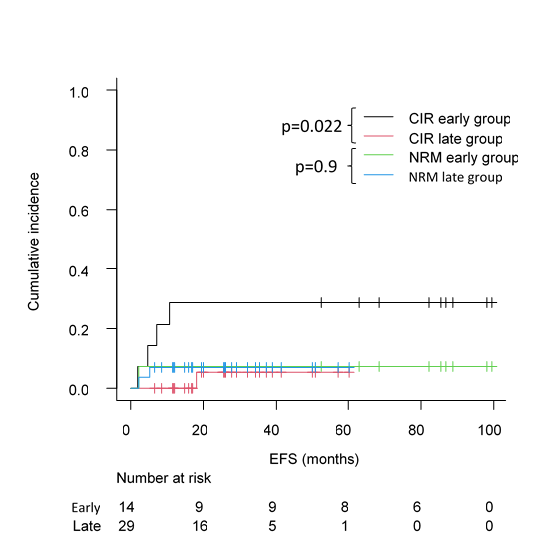
**Supplemental figure 6:** CIR of allotransplanted patients with *NPM1*mut and *FLT3*-ITD high ratio in each time period.


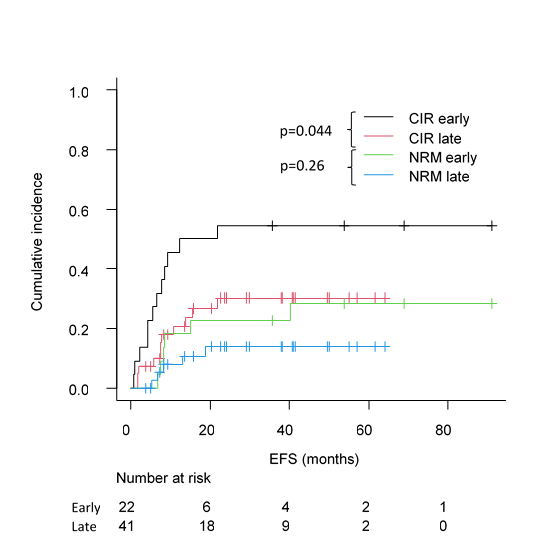


**Supplemental Table 2:** Patient characteristics of CETLAM AML-12 *NPM1*wt/*FLT3*mut patients

|  | 2012-2015 (n=33) | | 2016-2020 (n=42) | p |
| --- | --- | --- | --- | --- |
| Female gender n (%) | 20 (61) | 24 (57) | | 0.8 |
| Median Age (range) | 53 (21-69) | 52 (22-69) | | 0.6 |
| Median WBC x10^9^/L (range) | 42 (2-313) | 22 (0.4-304) | | 0.08 |
| Median % BM blasts (range) | 82 (30-96) | 75 (21-98) | | 0.1 |
| Cytogenetics (MRC 2010), n(%) |  |  | | 0.9 |
| Favourable | 3 (9) | 4 (9) | |  |
| Intermediate | 26 (79) | 31 (74) | |  |
| Adverse | 3 (9) | 5 (12) | |  |
| No metaphases | 1 (3) | 2 (5) | |  |
| *FLT3*-ITD n (%) | 32 (97) | 33 (79) | | 0.9 |
| Low ratio n | 7 | 7 | |  |
| High ratio n | 25 | 26 | |  |
| *FLT3*-TKD^#^ n (%) | 1 (3) | 8 (19) | | - |
| *FLT3*-other n (%) | - | 1 (2) | |  |
| CR after induction n (%) | 24 (73) | 30 (71) | | 0.9 |
| Nº cycles to CR (1) n (%) | 19 (79) | 27 (90) | | 0.3 |
| AlloHSCT n (%) | 19 (58) | 31 (74) | | 0.3 |

BM: bone marrow, CR1: first complete remission, NA: not applicable, WBC: leucocyte count

# TKD mutations detected only from late 2015

**Supplemental Figure 7**: Outcome distribution at last follow-up in *FLT3*mut/*NPM1*wt patients

c

b

a

*Late (2016-2020)*

*Early (2012-2015)*

^a^2 patients harboured CBF rearrengements (1 inv(16) and 1 t(8;21)), 1 patients received autologous transplant per center decission
^b^2 patients with t(8;21)
^c^1 patient with inv(16)

**
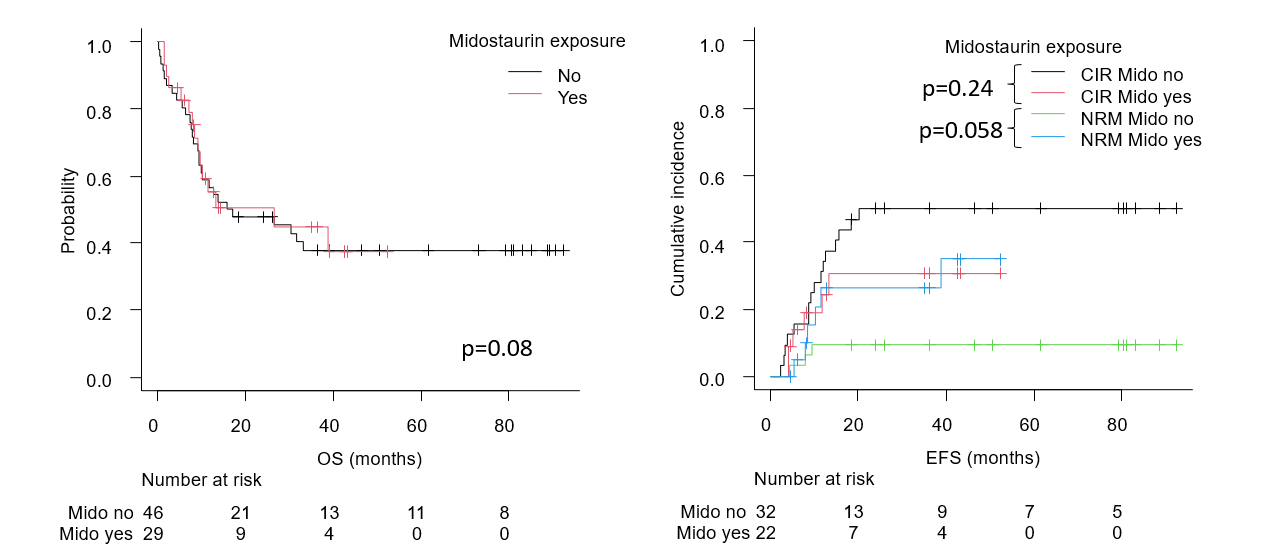
Supplemental Figure 8:** Midostaurin effect in the subset of *FLT3*mut patients without *NPM1* mutation

**Supplemental figure 9:** Risk of relapse of *FLT3*mut/*NPM1*wt patients. CIR: cumulative incidence of relapse, NRM: non-relapse mortality


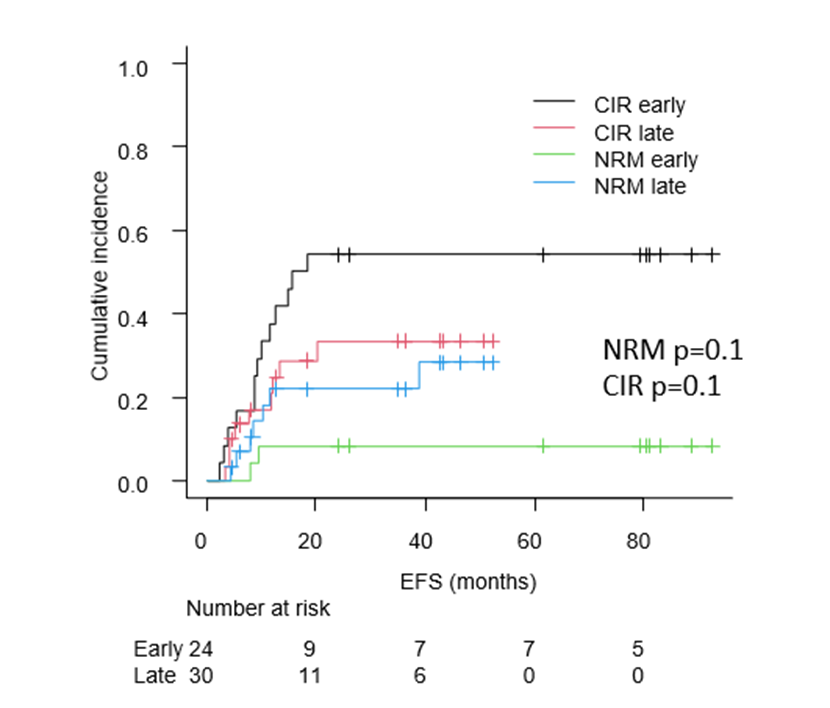


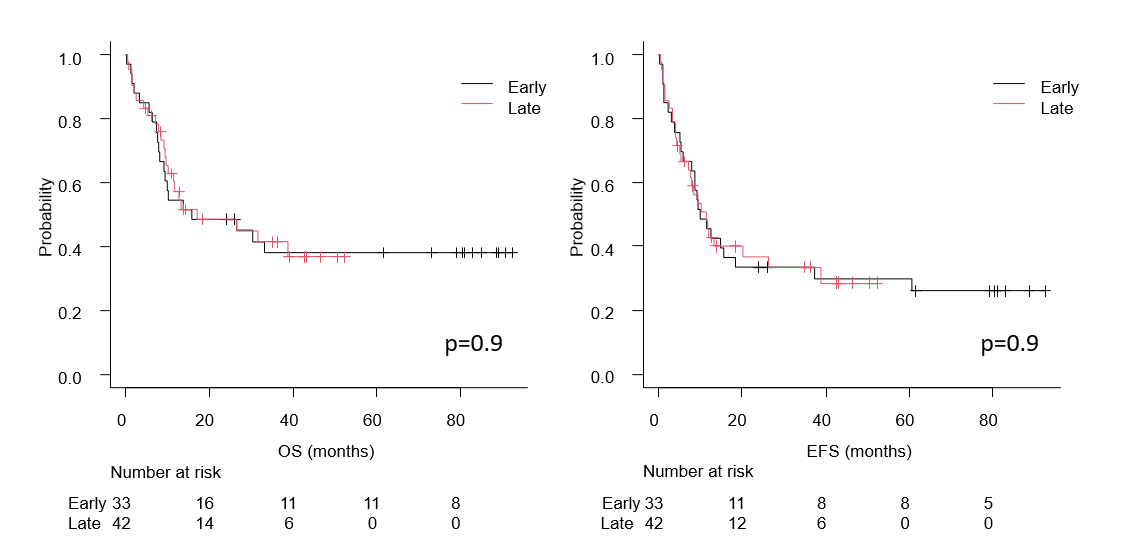
**Supplemental figure 10:** OS and EFS of *FLT3*mut/*NPM1*wt patients
